# Outcomes of Concurrent Ventral Hernia Repair and Cholecystectomy Compared to Ventral Hernia Repair Alone

**DOI:** 10.7759/cureus.45699

**Published:** 2023-09-21

**Authors:** Timothy P Becker, Ben Duggan, Varun Rao, Genaro Deleon, Kevin Pei

**Affiliations:** 1 General Surgery, Indiana University School of Medicine, Indianapolis, USA; 2 Neurological Surgery, Indiana University School of Medicine, Indianapolis, USA; 3 General Surgery, Parkview Health, Fort Wayne, USA

**Keywords:** acs-nsqip, outcomes, surgery, cholecystectomy, hernia

## Abstract

Introduction

It has been suggested that hernia repair with concomitant cholecystectomy increases the risk of postoperative complications due to potential mesh contamination. This study compares postoperative outcomes and complications between patients who underwent ventral hernia repair (VHR) with and without concomitant cholecystectomy (CCY).

Methods

Using the American College of Surgeons National Surgical Quality Improvement Program (ACS NSQIP) database, from 2005 to 2019, we queried patients who underwent ventral hernia repairs using the current procedural terminology (CPT) codes 49652-49657 (laparoscopic) and 49560-49566 (open), with or without cholecystectomy. The ACS NSQIP is a prospective, systematic study of patients who underwent major general surgical procedures aggregating data from over 200 hospitals. Cases involving additional concomitant procedures were excluded. Primary outcomes of interest were 30-day mortality, length of stay, readmission, return to operating room (OR), and postoperative complications. The odds ratio for primary outcomes was calculated using multivariable binomial logistic regression to control for patient risk factors.

Results

In total, 167586 cases were identified, 165,758 ventral hernia repairs alone, and 1,828 ventral hernia repairs with concomitant cholecystectomy. There was no difference in 30-day mortality, length of stay, readmission, return to the operating room, or postoperative complications between groups. Patients who underwent simultaneous VHR/CCY when compared to those who had VHR alone, had no differences in the rate of surgical site infections (1.86% vs. 1.97%, P = 0.57) or sepsis (0.82% vs. 0.41%, P = 0.10).

Conclusion

In a large national sample, there is no significant difference in postoperative outcomes, specifically infection-related complications, when comparing VHR along with concurrent VHR/CCY. Our findings suggest no increased risks for patients undergoing concurrent ventral hernia repair and cholecystectomy. Hence, surgeons might consider this combined approach to offer the best value-based care, especially when it could eliminate the need for a second operation and the risk of infection is low. Prospective studies with more procedural-specific information for hernia repairs and indications for cholecystectomy are needed however it is likely safe to perform both procedures during the same setting in cholecystectomy cases lacking signs of acute infection.

## Introduction

This article was previously presented as an abstract at the 2022 American College of Surgeons Clinical Congress on October 18, 2022. The presented abstract was published in volume 235 of the Journal of American College of Surgeons. 

Ventral hernia repair (VHR) represents a clinical challenge for surgeons and a significant healthcare burden; there are an estimated 300,000 VHRs completed each year in the United States, with a projected annual increase of 11,000 VHRs [1]. Similarly, gallstones are one of the most common diagnoses in the US, affecting 0.60-1.39% of the general population per year and contributing substantially to healthcare spending [2,3]. The clinical presentation varies considerably from asymptomatic stones to symptomatic cholelithiasis to more severe disease states such as acute cholecystitis, pancreatitis, and cholangitis. Although asymptomatic cholelithiasis is often managed without surgery, concomitant cholecystectomy is a reasonable option for patients undergoing unrelated abdominal surgery [4]. Additionally, the most common indication for cholecystectomy is symptomatic cholelithiasis [5,6]. As a result, it is not uncommon to have cholecystectomy (CCY) performed in cases where the primary procedure is a VHR [7].

The field of laparoscopic surgery has progressed significantly from when the first laparoscopic cholecystectomy (LC) was performed in 1985 [8]. Laparoscopic cholecystectomy has been performed concomitantly with numerous procedures, including appendectomy, splenectomy, hysterectomy, sleeve gastrectomy, and bile duct exploration [9-13]. Despite this, surgeons have debated the safety of combining cholecystectomy with hernia repairs primarily due to the possibility of mesh infections. This is an understandable concern as even in cases without acute infection, studies found 9-42% of elective cholecystectomy identified culture-positive bile [14,15]. 

While a reasonable concern, it is unknown from large cohort studies whether increased mesh infections in the setting of concomitant cholecystectomy are realized. This study compared postoperative outcomes and complications between patients who underwent ventral hernia repair with and without concomitant cholecystectomy. We hypothesized that patients undergoing ventral hernia repair with concomitant cholecystectomy would have no significant difference in postoperative outcomes compared to those undergoing ventral hernia repair alone.

## Materials and methods

Data source 

Data was obtained retrospectively from the American College of Surgeons National Surgery Quality Improvement Program (ACS NSQIP). The ACS NSQIP is a prospective, systematic study of patients who underwent major general surgical procedures aggregating data from over 200 hospitals. In participating hospitals, a data coordinator collects preoperative patient characteristics, intraoperative case details, and 22 uniformly defined postoperative adverse outcomes for 30 days after the procedure. Risk and outcome variables follow strict criteria and coordinators are trained on all study definitions. Follow-up information is obtained through phone calls, letters, and review of medical records. All NSQIP patients are risk-adjusted and case-mix-adjusted for comparison to ensure a standardized comparison of outcomes across different institutions. Institutional Review Boards provided an exemption for this study protocol.

Study design

This retrospective cohort study evaluated ASC-NSQIP cases between the years 2005 to 2019 (inclusive). We queried patients who underwent ventral hernia repairs using the current procedural terminology (CPT) codes for laparoscopic ventral hernia repairs (CPT 49652-49657) and open ventral hernia repairs (CPT 49560-49566), with or without concomitant cholecystectomy (CPT 47562-47564, 47600, 47605 & 47610). Cases involving any additional procedures were excluded so as to not confound the data. The focus was exclusively on cases where VHR was listed as the primary procedure, given its common association with concomitant procedures, and acknowledging previous studies have already examined ACS NSQIP cases involving incidental hernia repairs performed during CCY.

Data gathered included over 50 preoperative patient characteristics, intraoperative case details, and 22 postoperative adverse outcomes. Specific outcomes of interest analyzed include 30-day clinical outcomes, mortality, length of stay, readmission, and return to operating room (OR). Given that a theoretical increase in the risk of infection is the principal concern behind performing both procedures concomitantly the specific 30-day clinical outcomes examined include wound occurrences (superficial, deep, or organ/space surgical site infection and/or wound dehiscence) and sepsis or septic shock. Parkview IRB exempted this study from review. 

Statistical analysis

We examined and controlled for more than 20 select preoperative patient characteristics, including demographics and comorbidities using a multivariable logistic regression. The odds ratio and significance values for primary outcomes of interest were calculated after controlling for patient variables and risk factors. Specific preoperative patient characteristics controlled for this can be found in Table 1. P value significance and all confidence intervals were set at 0.05. Analysis was performed using R Core Team, version 3.6.1 (R Foundation for Statistical Computing, Vienna, Austria).

**Table 1:** Patient demographics, pre-operative risk factors, and co-morbidities. NSQIP = National Surgical Quality Improvement Program, BMI = Body Mass Index, Hx CHF = History of Congestive Heart Failure, Hx COPD = History of Chronic Obstructive Pulmonary Disease, Pre-op = preoperative, ASA = American Society of Anesthesiologist, SIRS = Systemic Inflammatory Response Syndrome

Surgery	Hernia, N = 165,758	Hernia + Cholecystectomy, N = 1,828	P - value
Mean Age (years)	40	41	0.3
SEX			<0.001
Female (n, %)	88,859 (54%)	1,148 (63%)	
Male (n, %)	76,833 (46%)	680 (37%)	
RACE			<0.001
White (n, %)	121,339 (73%)	1,156 (64%)	
American Indian or Alaska Native (n, %)	779 (0.5%)	6 (0.3%)	
Asian (n, %)	1,479 (0.9%)	20 (1.1%)	
Asia or Pacific Islander (n, %)	31 (<0.1%)	0 (0%)	
Black or African American (n, %)	18,795 (11%)	181 (9.9%)	
Black, Not of Hispanic Origin (n, %)	502 (0.3%)	0 (0%)	
Hispanic, Black (n, %)	4 (<0.1%)	0 (0%)	
Hispanic, Color Unknown (n, %)	167 (0.1%)	0 (0%)	
Hispanic, White (n, %)	99 (<0.1%)	0 (0%)	
Native Hawaiian or Pacific Islander (n, %)	285 (0.2%)	5 (0.3%)	
﻿﻿Unknown/Not Reported	16,340 (9.9%)	425 (23%)	
Mean BMI (kg/m^2^)	32	32	0.3
DIABETES STATUS			0.15
Insulin (n, %)	7,795 (4.7%)	101 (5.5%)	
NO (n, %)	139,612 (84%)	1,516 (83%)	
Non-Insulin (n, %)	17,018 (10%)	201 (11%)	
Oral (n, %)	1,333 (0.8%)	10 (0.5%)	
Current Smoking (n, %)	30,855 (19%)	281 (15%)	<0.001
DYSPNEA			
Aa Rest (n, %)	549 (0.3%)	4 (0.2%)	<0.001
Moderate Exertion (n, %)	8,969 (5.4%)	139 (7.6%)	
NO (n, %)	156,240 (94%)	1,685 (92%)	
FUNCTIONAL HEALTH STATUS			0.9157
Independent (n, %)	162,958 (98%)	1,795 (98%)	
Partially Dependent (n, %)	1,510 (0.9%)	19 (1.0%)	
Totally Dependent (n, %)	200 (0.1%)	2 (0.1%)	
Unknown (n, %)	1,090 (0.7%)	12 (0.7%)	
Ventilator Dependent (n, %)	57 (<0.1%)	0 (0%)	>0.9
Ascites (n, %)	719 (0.4%)	2 (0.1%)	0.036
Hx CHF (n, %)	728 (0.4%)	13 (0.7%)	0.082
Hx COPD (n, %)	7,434 (4.5%)	83 (4.5%)	0.9
Medication for Hypertension (n, %)	76,814 (46%)	825 (45%)	0.3
Pre-op Renal Failure (n, %)	233 (0.1%)	5 (0.3%)	0.12
Dialysis (n, %)	1,225 (0.7%)	13 (0.7%)	>0.9
Disseminated Cancer (n, %)	785 (0.5%)	13 (0.7%)	0.14
Open Wound/Infection (n, %)	1,076 (0.6%)	8 (0.4%)	0.3
Chronic Steroid Use (n, %)	5,401 (3.3%)	50 (2.7%)	0.2
>10% Loss Bodyweight in Last 6 mo (n, %)	524 (0.3%)	11 (0.6%)	0.031
Bleeding Disorder (n, %)	3,972 (2.4%)	45 (2.5%)	0.8
Pre-op Transfusion (n, %)	142 (<0.1%)	3 (0.2%)	0.2
ASA CLASS			0.02
1. No Disturb (n, %)	12,006 (7.2%)	120 (6.6%)	
2. Mild Disturb (n, %)	81,871 (49%)	852 (47%)	
3. Severe Disturb (n, %)	67,122 (41%)	788 (43%)	
4. Life Threat (n, %)	4,519 (2.7%)	64 (3.5%)	
5. Moribund (n, %)	29 (<0.1%)	0 (0%)	
None Assigned (n, %)	184 (0.1%)	4 (0.2%)	
PRE-OP SEPSIS			<0.001
None (n, %)	162,708 (98%)	1,757 (96%)	
Sepsis (n, %)	228 (0.1%)	17 (0.9%)	
Septic (n, %)	6 (<0.1%)	0 (0%)	
Septic Shock (n, %)	48 (<0.1%)	2 (0.1%)	
SIRS (n, %)	2,509 (1.5%)	48 (2.6%)	

## Results

In total, 167,586 cases were identified, of them, 165,758 were of ventral hernia repairs alone, and 1,828 ventral hernia repairs with concomitant cholecystectomy. Out of the 1,828 cases of ventral hernia repairs, 1,821 of concomitant cholecystectomy were performed using a laparoscopic approach. No difference was found in the average age of patients undergoing VHR alone vs. VHR with concomitant CCY (40 vs. 41 years, P = 0.3). Patients who underwent concomitant procedures were more likely to be female (63% vs. 53%, P < 0.001). Detailed patient demographics, pre-operative risk factors, and co-morbidities are summarized in Table 1.

After controlling for patient risk factors, we found no difference in our primary outcome measures. No statistically significant difference was found between groups in 30-day mortality, length of stay, readmission, or return to operating room (OR) between ventral hernia repairs alone and concomitant hernia repair with cholecystectomy. No difference was found in the rates of surgical site infection between VHR alone vs. VHR with concomitant CCY (1.97% vs. 1.87%, P =0.46). Specific outcomes, odds ratio, and P - values are listed in Table 2.

**Table 2:** Comparison of outcomes between standalone hernia repairs and hernia repairs with concomitant cholecystectomy using multivariate logistic regression. SSI = Surgical Site Infection.

Surgery	Hernia (N = 165,758)	Hernia + Cholecystectomy (N = 1,828)			
Complications	n (%)	n (%)	Estimated Odds Ratio (95% CI)	P Value	
30-day Mortality	431 (0.26%)	4 (0.22%)	0.64 (0.23, 1.84)	0.392991	
Readmission Related to Procedure	4067 (2.45%)	49 (2.68%)	1.01 (0.76, 1.35)	0.922519	
Return to OR	2416 (1.46%)	22 (1.2%)	0.81 (0.53, 1.24)	0.336518	
Any SSI	3262 (1.99%)	34 (1.86%)	0.88 (0.62, 1.24)	0.460129	
Superficial SSI	2050 (1.24%)	22 (1.20%)	0.91 (0.60, 1.40)	0.672638	
Deep Incisional SSI	619 (0.37%)	2 (0.11%)	0.29 (0.07, 1.18)	0.08259	
Organ Space SSI	638 (0.38%)	10 (0.55%)	1.29 (0.68, 2.42)	0.428615	
Wound Disruption	381 (0.23%)	6 (0.33%)	1.46 (0.64, 3.27)	0.363325	
Sepsis	683 (0.41%)	15 (0.82%)	1.61 (0.92, 2.72)	0.083424	

## Discussion

Our study determined that concomitant CCY and VHR compared to VHR alone, was not associated with worse 30-day outcomes. Furthermore, it was encouraging to find that concomitant procedures did not result in an increased rate of readmission or longer postoperative stays. The types of hernias include a wide range of hernias including reducible or incarcerated ventral, incisional, Spigelian, epigastric, femoral, and paraoesophageal. 

By definition, exposure to the biliary tract changes a hernia repair from a clean procedure to a clean-contaminated case. Although there is a theoretical risk of infection, there are numerous studies noting the safety of concomitant cholecystectomy with various types of hernia repairs. Transabdominal pre-peritoneal inguinal hernia repair has been shown to be safe and not associated with increased mesh infection when performed simultaneously with laparoscopic cholecystectomy in several recent case series [16-18]. Similar findings are reported in studies of concomitant CCY with subtypes of VHRs, including paraumbilical, umbilical, and those requiring abdominal wall reconstruction, demonstrating effectiveness and no increase in wound morbidity [7,19,20]. Large studies on concomitant VHR and CCY are limited, largely due to the low volume of cases. However, it is possible that, in practice, improvements in CCY techniques, perioperative management, and equipment have minimized the risk of contamination. As laparoscopic techniques are advanced by surgeons, the combination of procedures previously performed separately will likely continue to rise. One additional benefit to concomitant procedures is that it also potentially eliminates the need for a second operation which is an important consideration for delivering the best value-based care. While the NSQIP database does not include data on operation cost, other studies have found performing hernia repairs and LC concomitantly is substantially cheaper than performing both as separate procedures [16].

Our study had several limitations. Due to the retrospective nature and lack of a procedural-specific database for hernia repairs, potentially important variables could not be considered, such as mesh material, antibiotics used, and hernia size. While hernia-specific details may impact the outcomes, it is more likely that patient factors, including comorbid conditions, contributed more significantly. The database used did not include ICD codes for concomitant procedures meaning the indication for performing the cholecystectomy was unknown. Surgeon selection bias may have been introduced where surgeons avoided concomitant cholecystectomy in high-risk patients, which might explain why the rate of surgical site infection was lower for patients who underwent concomitant procedures. Although we found no evidence of worse outcomes for CCY performed concomitantly with VHR; further prospective randomized studies are necessary to understand the true risk-benefit of concomitant procedures. 

## Conclusions

In a large national sample, there is no significant difference in postoperative outcomes, and specifically infection-related complications, when comparing ventral hernia repair with ventral hernia and concurrent cholecystectomy. Our findings suggest no increased risks for patients undergoing concurrent ventral hernia repair and cholecystectomy. Hence, surgeons might consider this combined approach to offer the best value-based care, especially when it could eliminate the need for a second operation and the risk of infection is low. Prospective studies with more procedural-specific information for hernia repairs and indications for cholecystectomy are needed however, it is likely safe to perform both procedures during the same setting in cholecystectomy cases lacking signs of acute infection.
